# Single-Crystalline InGaAs Nanowires for Room-Temperature High-Performance Near-Infrared Photodetectors

**DOI:** 10.1007/s40820-015-0058-0

**Published:** 2015-09-21

**Authors:** Huang Tan, Chao Fan, Liang Ma, Xuehong Zhang, Peng Fan, Yankun Yang, Wei Hu, Hong Zhou, Xiujuan Zhuang, Xiaoli Zhu, Anlian Pan

**Affiliations:** Key Laboratory for Micro-Nano Physics and Technology of Hunan Province, School of Physics and Electronics, and State Key Laboratory of Chemo/Biosensing and Chemometrics, Hunan University, Changsha, 410082 People’s Republic of China

**Keywords:** InGaAs, Nanowire, Near-infrared, Photodetector

## Abstract

InGaAs is an important bandgap-variable ternary semiconductor which has wide applications in electronics and optoelectronics. In this work, single-crystal InGaAs nanowires were synthesized by a chemical vapor deposition method. Photoluminescence measurements indicate the InGaAs nanowires have strong light emission in near-infrared region. For the first time, photodetector based on as-grown InGaAs nanowires was also constructed. It shows good light response over a broad spectral range in infrared region with responsivity of 6.5 × 10^3^ A W^−1^ and external quantum efficiency of 5.04 × 10^5^ %. This photodetector may have potential applications in integrated optoelectronic devices and systems.

**Electronic supplementary material:**

The online version of this article (doi:10.1007/s40820-015-0058-0) contains supplementary material, which is available to authorized users.

## Introduction

One-dimensional semiconductor nanowires have attracted considerable attention as unique building blocks for various interesting applications in integrated electronic and optoelectronic devices and systems, such as nanoscale lasers, field-effect transistors, solar cells, and photodetectors [1–8]. Among them, nanowire-based photodetectors have aroused wide interests as potential key functional units in on-chip information communication and processing [9, 10]. Although much work has been devoted to investigating nanowire-based photodetectors in the past few years [10–16], most of the reported nanowire detectors are limited in the visible and ultraviolet spectral regions [9, 11, 13, 14], and little work is conducted in the infrared region [15, 16]. III–V semiconductor nanowires with narrow bandgap are considered as promising candidates for constructing infrared photodetectors. To date, infrared photodetectors based on InAs, InPAs, and InGaSb nanowires have been reported [8, 10, 16, 17].

As an important III–V ternary semiconductor, InGaAs are expected to have potential bandgap tunability from the near-infrared (NIR) to mid-infrared (MIR) region (0.35 ≤ *E*_g_ ≤ 1.42 eV). Due to its tunable bandgap, as well as high electron mobility and small leakage current, In_x_Ga_1−x_As nanowires have been widely used in optoelectronic devices, such as NIR emission lasers, photovoltaics, and field-effect transistors [18–20]. Till now photodetectors based on InGaAs quantm dots and films have been fabricated. However, the performances of these devices are relatively poor. It is desirable to fabricate InGaAs photodetectors with high performance based on other nanomaterials. To the best of our knowledge, there is no report on room-temperature infrared photodetector based on InGaAs nanowires.

In this work, InGaAs nanowires were first synthesized via a simple chemical vapor deposition (CVD) method. Raman and photoluminescence measurements illustrate that the as-grown nanowires are high-quality single crystals. More importantly, integrated infrared photodetectors were constructed by using these as-grown nanostructures. The achieved devices exhibit good photoresponse in a broad spectral range from 1100 to 2000 nm, in which the responsivity (R) and the external quantum efficiency (EQE) are comparable with those infrared photodetectors based on other III–V nanostructures at room temperature [8, 16]. Our results may possess important applications in integrated photonics and optoelectronics devices.

## Experimental

CVD method was used to grow InGaAs nanowires. Briefly, InAs power (Alfa Aesar, 99.99 %) and GaAs power (Alfa Aesar, 99.99 %) were mixed equivalently and placed into the heating zone of the furnace. Silicon wafers coated with 5-nm-thick gold film were placed downstream with a distance of 24 cm away to the center of tube furnace to collect the deposited InGaAs nanowires. Before heating Ar mixed with 10 % H_2_ gas flowed through the horizontal quartz tube at a rate of 45 sccm and the pressure maintained at 3 Torr. The temperature of the powder sources was set to 860 °C and that of the substrate was approximately 520 °C (schematic diagram of the experimental setup and the temperature gradient in the furnace see Fig. S1). After 90 min of growth at 860 °C, the temperature was reduced naturally to room temperature.

The morphology of the as-prepared sample was characterized by field emission scanning electron microscopy (FE-SEM, Hitachi S-4800). The phase characterization was identified using XRD (Rigaku D/Max 2500). Transmission electron microscopy (TEM, Tecai F20) combined with energy-dispersive X-ray spectroscopy (EDX) was used to investigate the microstructure and elemental composition. Raman spectrum was performed by a µ-Raman (WITec alpha-300) system excited with a 488 nm argon ion laser. The photoluminescence (PL) measurements were carried out on a home-built IR micro-PL setup. The samples were excited by a passively mode-locked Ti:sapphire laser. (Spectra Physics Tsunami, 800 nm, 150 fs pulse duration, 80 MHz repetition rate.) The PL signal was collected and detected using a spectrometer (HORIBA iHR 550) equipped with a liquid nitrogen-cooled InGaAs photodiode detector (1300–2300 nm). The current–voltage (*I*–*V*) characteristics of the photodetectors were measured using a Keithley 4200.

## Results and Discussion

**Fig. 1:**
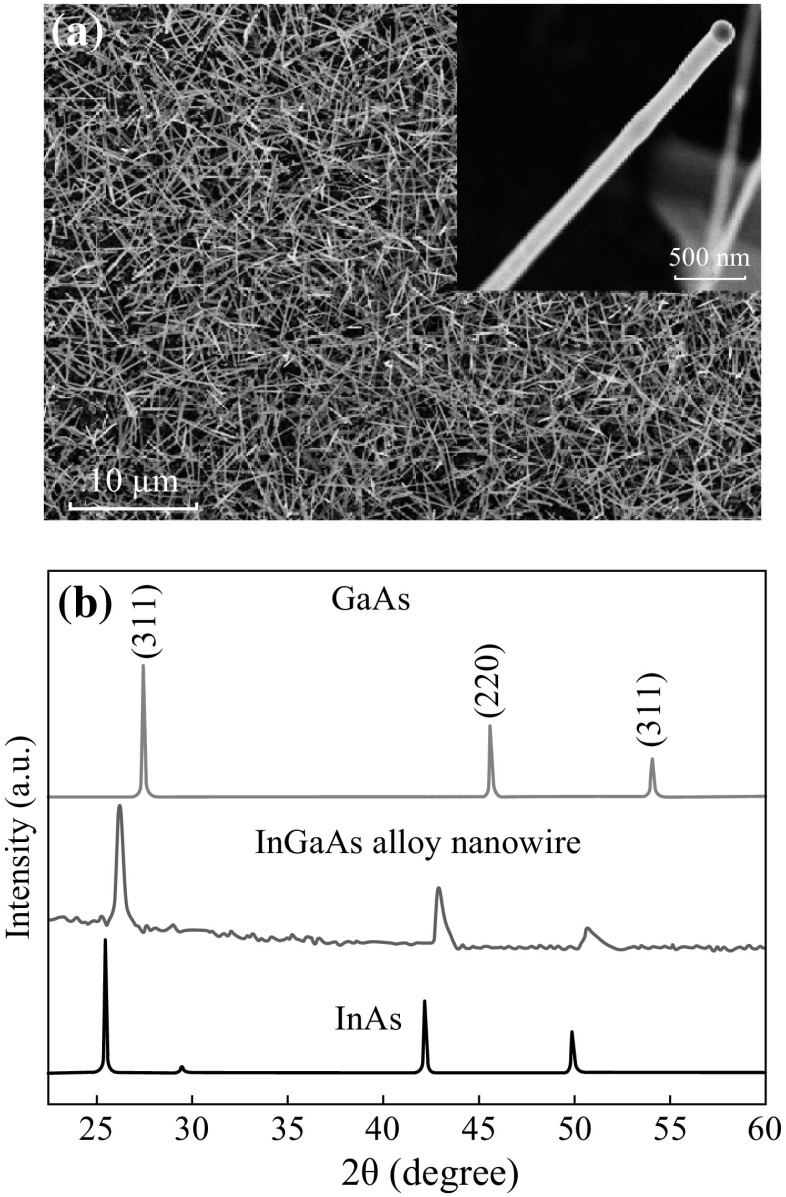
**a** Typical SEM image of the morphology of the as-grown nanowires. **b** XRD patterns of the as-deposited alloy nanowires

The typical SEM image of the as-grown sample is shown in Fig. 1a. The nanowires with uniform diameter and tens of micrometers length were deposited in high yields on the Si substrate. The inset of Fig. 1a shows a high-magnification SEM image of a typical nanowires with a diameter of about 150 nm. Figure 1b represents the XRD pattern of the as-deposited alloy nanowires. For contrasting study, XRD patterns of pure InAs (bottom, JCPDS No. 88-2489) and GaAs (top, JCPDS No. 89-3314) are also plotted. All the diffraction peaks located between those of zinc blende InAs (bottom, JCPDS No. 88-2489) and GaAs (top, JCPDS No. 89-3314) single crystals are clearly seen, confirming the as-grown sample is an InGaAs alloy with zinc blende crystallographic phase. There are no characteristic peaks from other oxides or crystalline impurities, suggesting the formation of high-purity InGaAs nanowires.

**Fig. 2:**
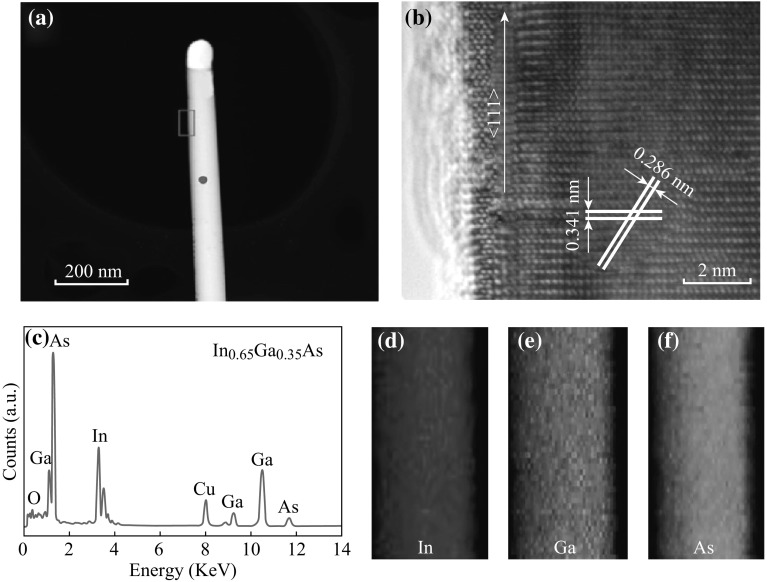
**a** TEM image of a representative single InGaAs nanowire. **b** HRTEM image taken from the pink rectangle of Fig. 2a. **c** Corresponding EDX profiles measured from the red position of the nanowire. **d**–**f** 2D elemental mapping for the three detected elements: In, Ga, and As, respectively

Figure 2a shows the TEM image of a representative nanowires. It can be seen that the surface of the nanowire is smooth, and there is a typical spherical catalytic particle at the tip of the nanowire evidenced by the typical metal-catalyzed vapor–liquid–solid (VLS) growth mechanism. Figure 2b displays the high-resolution TEM (HRTEM) image taken from the nanowire (the pink rectangle in Fig. 2a). It demonstrates that the nanowire has single-crystalline zinc blende (ZB) structure. The plane spacing is 0.341 and 0.286 nm, which is corresponding well to (111) and (200) lattice planes in the In-rich thin-film counterparts [21], respectively. Figure 2c illustrates the corresponding EDX spectra of the nanowire. The peaks of In, Ga, and As have strong intensities and their atomic ratio is close to 0.65:0.35:1, demonstrating that the composition of the achieved wires is In_0.65_Ga_0.35_As alloys (the detected Cu element originates from the copper grid). Figure 2d–f show the two-dimensional (2D) elemental mapping of this nanowire. As can be seen, In, Ga, and As are homogeneously distributed across the whole nanowire.

**Fig. 3:**
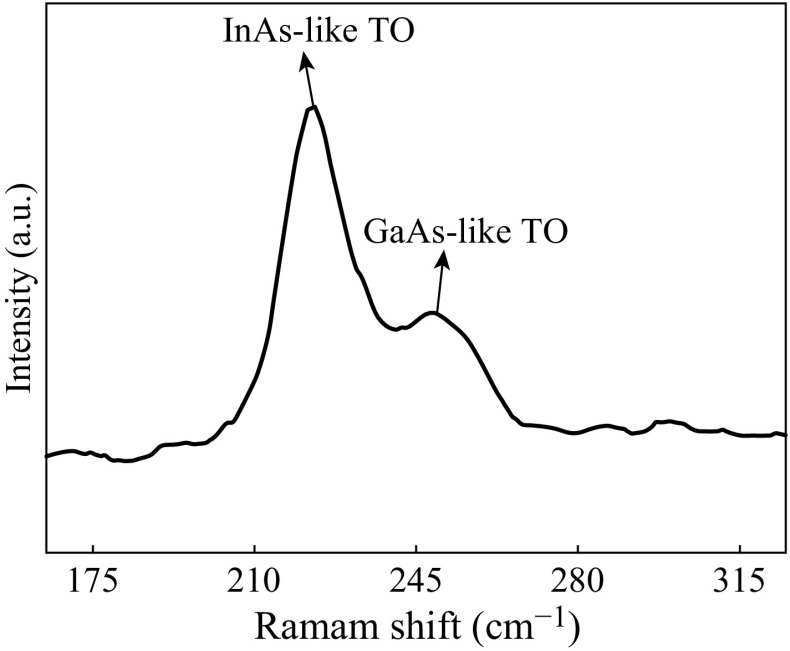
Raman spectrum of the In_0.65_Ga_0.35_As nanowires excited with a 488 nm argon ion laser

In order to further characterize the microstructural properties of the as-grown In_0.65_Ga_0.35_As nanowires, we have performed the Raman spectrum measurements. A typical Raman spectrum of the In_0.65_Ga_0.35_As nanowires is displayed in Fig. 3. Two phonon modes at 223 and 249 cm^−1^ are clearly seen, which is assigned to the InAs-like and GaAs-like transverse optical phonon modes, respectively. Regarding the peaks position of the two phonon modes, it can be found that the experimental data are in agreement well with literature values [22, 23]. This confirms that the achieved In_0.65_Ga_0.35_As nanowires have good compositional homogeneity and high-quality crystallization without the stacking disorder.

**Fig. 4:**
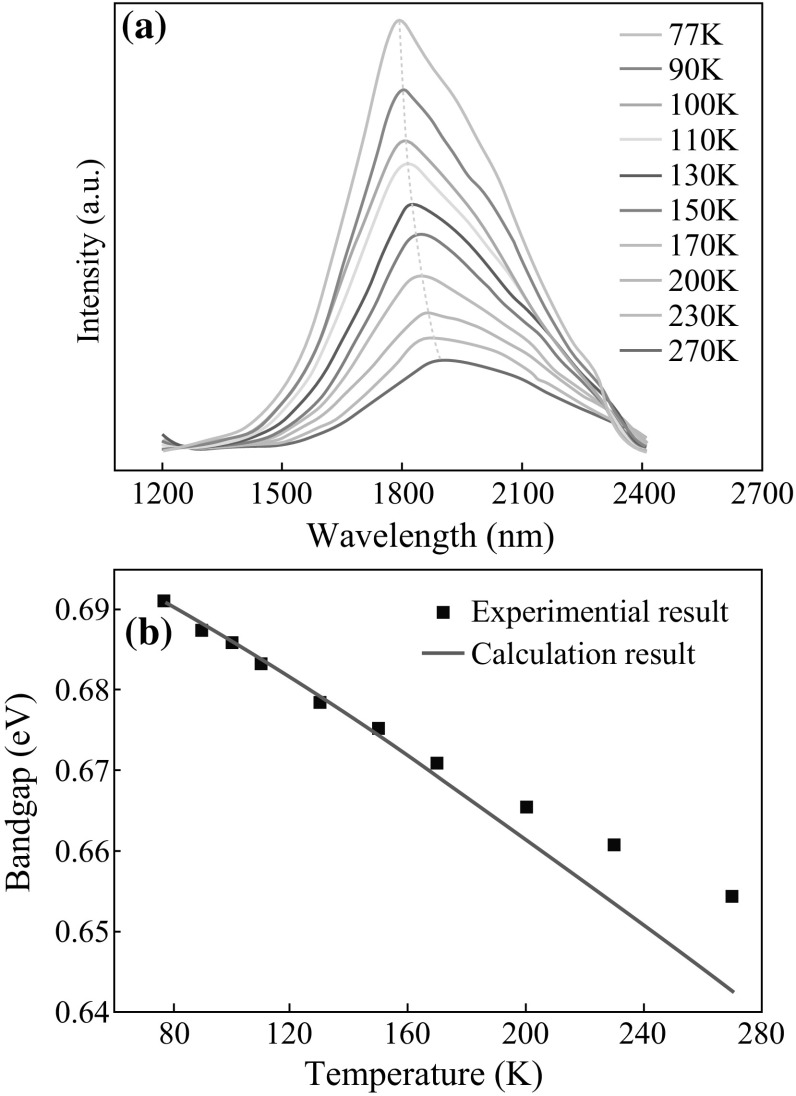
**a** Temperature-dependent PL spectra of the In_0.65_Ga_0.35_As nanowires under the same laser excitation, the dashed line shows the shift of the PL peak energy with changing the temperature. **b** Temperature-dependent bandgap of the achieved In_0.65_Ga_0.35_As nanowires

Figure 4a exhibits the temperature-dependent PL of the In_0.65_Ga_0.35_As nanowires excited with an 800-nm femtosecond laser at the temperature from 77 to 270 K. A strong PL emission band with the peak wavelength at 1794 nm can be observed at 77 K, which is well consistent with the band gap value of In-rich InGaAs alloy nanowires (0.69 eV) grown by MBE (Molecular Beam Epitaxy) at this temperature [24]. The temperature dependence of the band gap is a continuous function with the Varshni’s empirical relation $(E_{\text{g}} (T) = E_{\text{g}} (0{\text{K}}) - \alpha T^{2} /(T + \beta ))$ [25]. According to this equation, the fitted *α* and *β* are 3 × 10^−4^ eV K^−1^ and 105 K for the In_0.65_Ga_0.35_As (the calculated process sees Supporting Information). The measured experiment results obtained from PL peak position are consistent with the calculated band gap values as shown in Fig. 4b, which demonstrates that the observed PL is mainly from the bandedge emission of the In_0.65_Ga_0.35_. As nanowires without any observed defect-related emission bands. All of these results are in agreement well with the structural and composition investigations described above, further demonstrating the high-quality of the In_0.65_Ga_0.35_As nanowires.

**Fig. 5:**
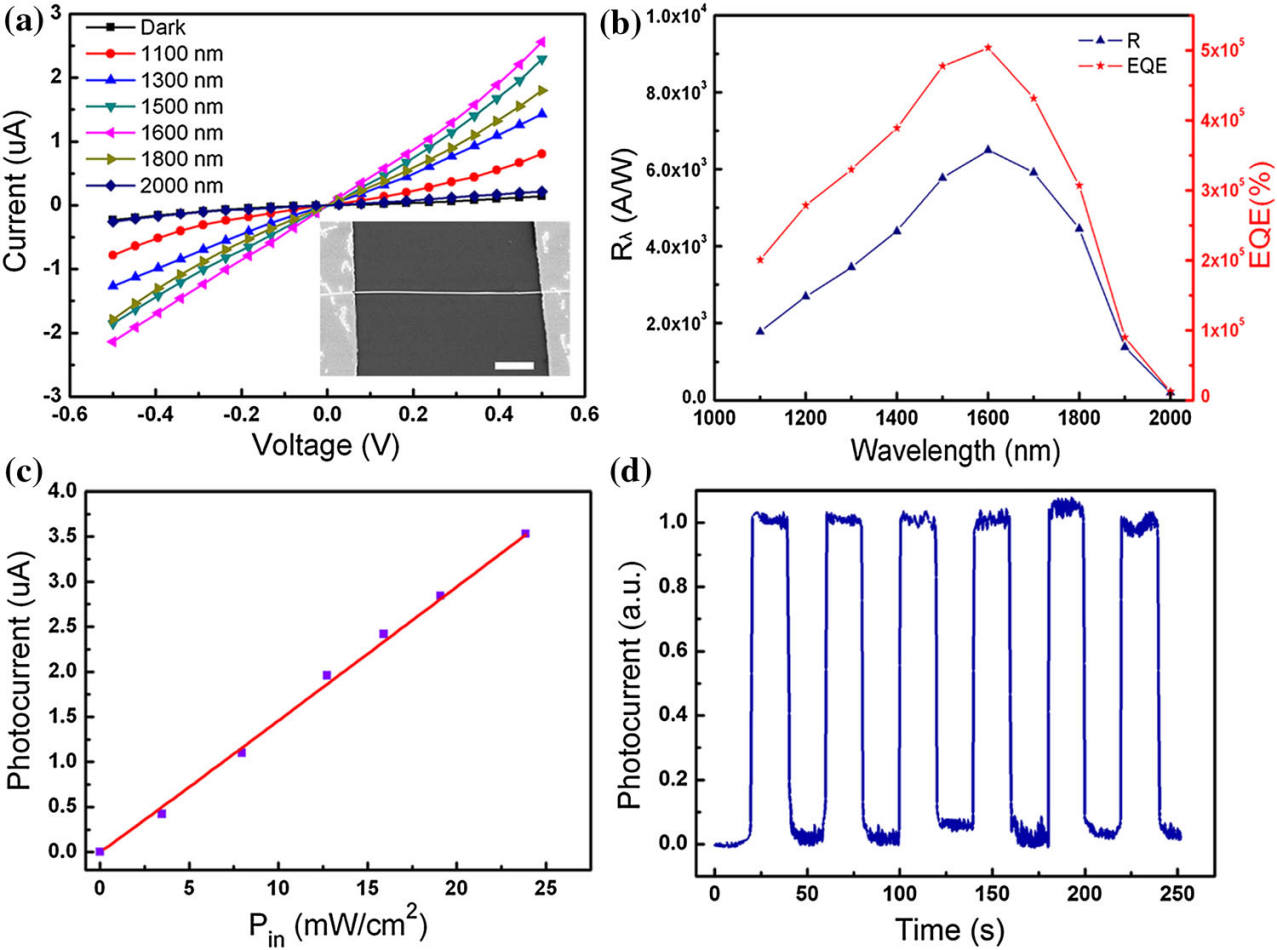
**a** *I*–*V* curves for a representative In_0.65_Ga_0.35_As nanowire photodetector under light illumination with different wavelengths (Pin = 15.8 mW cm^−2^) at room temperature, inset, SEM image of a fabricate photodetector, the scale bar is 2 μm. **b** The corresponding R and EQE versus incident wavelength of the photodetectors. **c** The photocurrent dependence of the incident light power density at a bias voltage of 0.5 V. **d** Time-dependent photocurrent response to 1600 nm light under 0.5 V bias

To shed light on studying the photoconductance properties of as-grown nanowires, photodetectors were constructed using these high-quality In_0.65_Ga_0.35_As alloy nanowires. The wires were first dispersed on a p-type Si substrate with 300-nm thickness of SiO_2_ layer. Photo-lithography was employed to define the source and drain pattern. Cr/Au (10/60 nm) source/drain (S/D) electrodes were made by metal evaporation and lift-off processes. A single InGaAs nanowire connected with the Cr/Au Schottky contact electrodes constitutes a typical M–S–M photodetector. Figure 5a shows the *I*–*V* curves of a representative nanowire photodetector (see the inset of the figure) with a wire diameter of *d* ∼ 150 nm and a channel length of *L* ∼ 10 μm, measured in the dark condition and under the illumination of a beam of a monochromatic light with the wavelength of 1100, 1300, 1500, 1600, 1800, and 2000 nm (light intensity *P*_in_ = 15.8 mW cm^−2^) at room temperature, respectively. At a fixed bias voltage of 0.5 V, it can be seen that the dark current is about 144 nA, and the largest photo-excited current reaching ~2.5 μA was recorded at the wavelength of 1600 nm. The curves show the electric conductance of the devices drastically increases under the light illumination of all the given wavelengths, suggesting good photoresponse ability of the device in near-infrared spectrum.

In order to get the intuitional result of the photoresponse, the calculated spectral responsivity (*R*_λ_) and external quantum efficiency (EQE) results of the In_0.65_Ga_0.35_As nanowire device for different wavelengths ranging from 1000 to 2000 nm are depicted in Fig. 5b. One can notice that the photoresponse increases gradually as the incident light wavelength changes from 1100 to 1600 nm and then decreases rapidly with the increase of light wavelength larger than 1600 nm. This indicates that the response spectrum is related to the energy band structure of In_0.65_Ga_0.35_As nanowires. The photons with larger energy than band gap are easily capable to excite the electron–hole pairs and therefore enhance the photoconductive sensitivity. These results demonstrate that the device based on In_0.65_Ga_0.35_As nanowires has good wavelength selectivity and high photosensitivity to infrared light in the NIR region from 1100 to 2000 nm.

R and EQE, two key parameters of a photodetector sensitivity to the incident light, can be expressed as [9, 13]

1 $$R = \frac{{I_{\text{ph}} }}{{P_{{}} S}}$$

2 $$EQE = R \times \frac{hc}{e\lambda },$$

where *I*_ph_ is the photocurrent, *P* is the light intensity, *S* is the effective illuminated area, *h* is Plank’s constant, *c* is the velocity of light, and *λ* is the incident light wavelength. The values of R and EQE at 1600 nm were calculated to be 6.5 × 10^3^ A W^−1^ and 5.04 × 10^5^ % at a bias voltage of 0.5 V, respectively. They are higher than those of conventional infrared photodetectors constructed with InGaAs quantum dots and thin films [26, 27].

It is well known that one-dimensional semiconducting nanowires own higher surface-to-volume ratio and can easily induce trap states at the nanowire surface. Those trap states will significantly affect the transport and photoconduction properties of the nanowire-based photodetectors. It was reported that In_0.65_Ga_0.35_As nanowire has higher density of surface electrons. Oxygen molecules can be easily adsorbed and combined with free electrons on the nanowire surface to form a low-conductivity depletion layer and result in increase of carrier densities [24]. Under NIR light illumination, the generated electron–hole pairs diffuse into the depletion region and then drift to the surface under the electric field in a radial direction and neutralize the adsorbed oxygen ions. This process releases back the captured free electrons and increases the free electron concentration. It was reported that the photoconductive gain can be expressed as *G* = τ/*T*_r_, where τ is the carrier lifetime and *T*_r_ is the transition time between electrodes. Because of the existence of an internal electric field, the recombination of carriers will be slowed down and the life time of the photocarrier (*τ*) will be prolonged [13]. For In_0.65_Ga_0.35_As nanowires with a proper radius of 150 nm, the active area of the carriers will be confined by one-dimensional structure and therefore the scattering and carrier trapping will reduce. This will result in a less *T*_r_ [28]. The nanowires in higher quality of defect-free single crystal can facilitate the transport of the carrier along the axis of the wires and therefore increase the photocurrent gain [29]. It can be concluded that the electron trapping at the nanowire surface and one-dimensional structure with higher quality are two key elements to affect the carrier drift for a photocurrent gain. In addition, the electrode distance and Schottky contact may also affect the photoresponse. The shorter distance of 10 μm between the two electrodes benefits to shorten the carrier transit time during the transport process. Moreover, the Schottky barriers in metal–semiconductor–metal (MSM) will reduce due to the photo-generated carriers and also benefits the electron injection [15, 30]. All of these elements can increase the photocurrent density effectively, and therefore lead to an enhancement of photoresponse.

Figure 5c displays the dependence of photocurrent on light intensity curves measured at a voltage of 0.5 V. It demonstrates a good linear relationship between the photocurrent and the light intensity, which is beneficial for application in light power detection. This indicates that the In_0.65_Ga_0.35_As nanowire is a typical photon-dependent resistor. There may exist complex processes of carrier generation, trapping, and recombination in the nanowires [31]. The higher photoresponse is mainly due to its inner photoelectrical effect [9]. Figure 5d shows the time-related reproducible response of the photocurrent to illumination at 1600 nm. One can see that the photodetector is stable under an optical power of 15.8 mW cm^−2^ and a bias voltage of 0.5 V. The response time and recovery time, defined as the time between 10 and 90 % of maximum photocurrent, is respective 70 and 280 ms (the detailed curves of photocurrent changing with time see Fig. S2). These values are comparable with those of InAs nanowire photodetectors [7].

## Conclusion

In summary, single-crystal In_0.65_Ga_0.35_As nanowires were synthesized by a simple CVD method. The nanowires have strong light emission in the near-infrared region. Photodetector based on as-grown nanowires was constructed and it exhibits a good photoresponse over a broad range from 1100 to 2000 nm. A higher responsivity of 6.5 × 10^3^ A/W and external quantum efficiency of 5.04 × 10^5^ % were observed. The photodetector in this work may have potential applications in integrated optoelectronic devices for infrared radiation imaging, sensing, and information communications.

## Electronic supplementary material

Below is the link to the electronic supplementary material.

Supplementary material 1 (DOCX 388 kb)
