# Supplementary material for: Single-Crystalline InGaAs Nanowires for Room-Temperature High-Performance Near-Infrared Photodetectors
Source: Nanomicro Lett. 2015 Sep 21;8(1):29–35. doi: 10.1007/s40820-015-0058-0 (PMC6223916; doi:10.1007/s40820-015-0058-0)
Supplement: Supplementary file 1 — Supplementary material 1 (DOCX 388 kb) [file 40820_2015_58_MOESM1_ESM.docx]

Supporting Information for

**High-performance Near-infrared Photodetector Based on Single-crystalline InGaAs Nanowires**

Huang Tan^＋^, Chao Fan^＋^, Liang Ma, Xuehong Zhang, Peng Fan, Yankun Yang, Wei Hu, Hong Zhou, Xiujuan Zhuang, Xiaoli Zhu*, Anlian Pan*

Key Laboratory for Micro-Nano Physics and Technology of Hunan Province, School of Physics and Microelectronic Science, and State Key Laboratory of Chemo/Biosensing and Chemometrics, Hunan University, Changsha 410082, People’s Republic of China

^＋^Authors contribute equally in the work

*** Corresponding authors. E-mail:** [anlian.pan@hnu.edu.cn](mailto:anlian.pan@hnu.edu.cn)**,** [zhuxiaoli@hnu.edu.cn](file:///F:\学院管理\Nano-micro%20letters%20杂志\稿件处理\2015\20151142\zhuxiaoli@hnu.edu.cn)


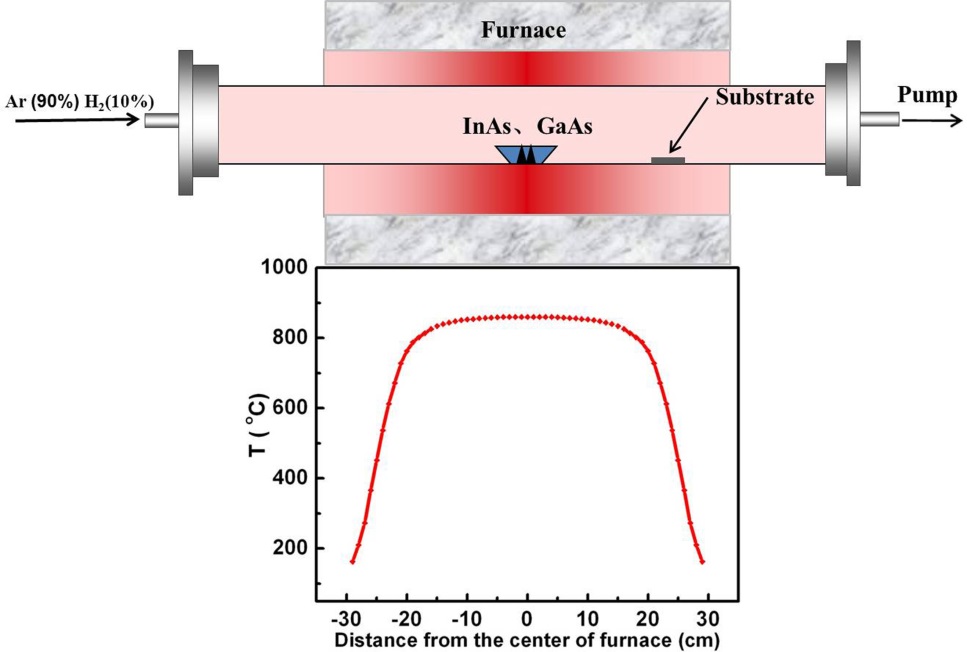


Fig. S1 Schematic diagram of the experimental setup and the temperature gradient in the furnace. The temperature profile of tube furnace was measured with thermocouples


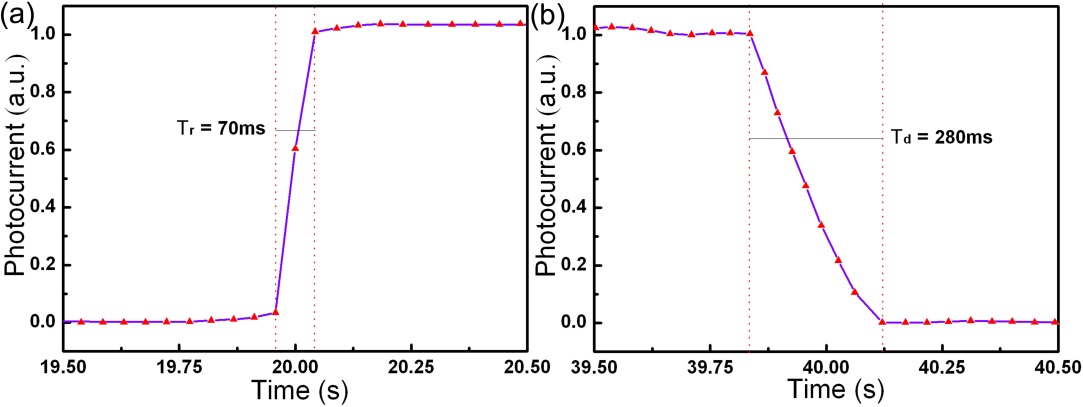


Fig. S2 (**a, b**) The detailed curves of photocurrent changing with time.

**The Temperature-dependent Bandgap of In_0.65_Ga_0.35_As NWs**

The temperature-dependent bandgap values of GaAs and InAs can be calculated from the two equation, respectively [1, 2]:

$E_{g}(T)=0.415-\frac{2.76\times10^{4}T^{2}}{T+83}$ (1)

$E_{g}(T)=1.515-\frac{5.5\times10^{4}T^{2}}{T+255}$ (2)

The theoretical bandgap of In*_x_*Ga_1-_*_x_*As can be given by the following equation:

$E_{g}(In_{x}Ga_{1-x}As)=E_{g}(InAs)x+E_{g}(GaAs)(1-x)-0.436x(1-x)$ (3)

The Varshni's empirical relation of a typical semiconductor [3]:

$E_{g}(T)=E_{g}(0K)-\alpha T^{2}/(T+\beta)$ (4)

According to these four equations, the bandgap of In_0.65_Ga_0.35_As can be calculated as:$E_{g}(In_{0.65}Ga_{0.35}As,T)=0.7008-\frac{3\times10^{4}T^{2}}{T+105}$ (5), as shown in Fig. 3b.

**References**

1. P. Kraisingdecha, M. Gal, Differential reflectance spectroscopy of GaAs/GaAlAs at elevated temperatures. Appl. Phys. Lett. **69**(10), 1355-1357 (1996). [doi:10.1063/1.117434](%20http:/dx.doi.org/10.1063/1.117434)
2. Z.M. Fang, K.Y. Ma, D.H. Jaw, R.M. Cohen, G.B. Stringfellow, Photoluminescence of InSb, InAs, and InAsSb grown by organometallic vapor phase epitaxy. J. Appl. Phys. **67**(11), 7034-7039 (1990). [doi:10.1063/1.345050](http://dx.doi.org/10.1063/1.345050)
3. Y.P. Varshni, Temperature dependence of the energy gap in semiconductors. Physica **34**(1), 149-154 (1967). [doi:10.1016/0031-8914(67)90062-6](http://dx.doi.org/doi:10.1016/0031-8914(67)90062-6)
